# Quantitative and histological assessment of maternal-fetal transmission of *Trypanosoma cruzi* in guinea pigs: An experimental model of congenital Chagas disease

**DOI:** 10.1371/journal.pntd.0006222

**Published:** 2018-01-24

**Authors:** Jatziri Torres-Vargas, Matilde Jiménez-Coello, Eugenia Guzmán-Marín, Karla Y. Acosta-Viana, Zaida E. Yadon, Eduardo Gutiérrez-Blanco, José Leonardo Guillermo-Cordero, Nisha J. Garg, Antonio Ortega-Pacheco

**Affiliations:** 1 Departamento de Salud Animal y Medicina Preventiva, Facultad de Medicina Veterinaria y Zootecnia, Universidad Autónoma de Yucatán, Mérida, Yucatán, México; 2 C.A. Biomedicina de Enfermedades Infecciosas y Parasitarias, Centro de Investigaciones Regionales "Dr. Hideyo Noguchi", Universidad Autónoma de Yucatán, Mérida, Yucatán, México; 3 Health Surveillance, Disease Prevention and Control, Pan American Health Organization, Duque de Caxias, Rio de Janeiro, Brazil; 4 Department of Microbiology & Immunology, University of Texas Medical Branch, Galveston, Texas, United States of America; Baylor College of Medicine, UNITED STATES

## Abstract

**Objective:**

We evaluated the effect of *Trypanosoma cruzi* infection on fertility, gestation outcome, and maternal-fetal transmission in guinea pigs (*Cavia porcellus*).

**Methods:**

Animals were infected with *T*. *cruzi* H4 strain (TcI lineage) before gestation (IBG) or during gestation (IDG). Tissue and sera samples of dams and fetuses were obtained near parturition.

**Results:**

All IBG and IDG dams were seropositive by two tests, and exhibited blood parasite load of 1.62±2.2 and 50.1±62 parasites/μl, respectively, by quantitative PCR. Histological evaluation showed muscle fiber degeneration and cellular necrosis in all infected dams. Parasite nests were not detected in infected dams by histology. However, qPCR analysis detected parasites-eq/g heart tissue of 153±104.7 and 169.3±129.4 in IBG and IDG dams, respectively. All fetuses of infected dams were positive for anti-parasite IgG antibodies and tissue parasites by qPCR, but presented a low level of tissue inflammatory infiltrate. Fetuses of IDG (vs. IBG) dams exhibited higher degree of muscle fiber degeneration and cellular necrosis in the heart and skeletal tissues. The placental tissue exhibited no inflammatory lesions and amastigote nests, yet parasites-eq/g of 381.2±34.3 and 79.2±84.9 were detected in IDG and IBG placentas, respectively. Fetal development was compromised, and evidenced by a decline in weight, crow-rump length, and abdominal width in both groups.

**Conclusions:**

*T*. *cruzi* TcI has a high capacity of congenital transmission even when it was inoculated at a very low dose before or during gestation. Tissue lesions, parasite load, and fetal under development provide evidence for high virulence of the parasite during pregnancy. Despite finding of high parasite burden by qPCR, placentas were protected from cellular damage. Our studies offer an experimental model to study the efficacy of vaccines and drugs against congenital transmission of *T*. *cruzi*. These results also call for *T*. *cruzi* screening in pregnant women and adequate follow up of the newborns in endemic areas.

## Author summary

Chagas disease is endemic in Latin America where the vector, hematophagous triatomine, is widely distributed. The exposure of women to *T*. *cruzi* may result in birth defects. However, congenital transmission of *Trypanosoma cruzi* from infected mothers to fetuses is scantily studied. Herein, we have developed a guinea pig model of congenital transmission of *T*. *cruzi* and evaluated the pathology in fetuses born to mothers that were exposed to *T*. *cruzi* before or during gestation. We also show high virulence of a *T*. *cruzi* DTU I strain and its capacity to invade fetal tissues even at a very low dose.

## Introduction

American trypanosomiasis, also known as Chagas disease, is caused by a flagellate protozoan *Trypanosoma cruzi* (*T*. *cruzi)*. The clinical course of Chagas disease is divided into the acute and chronic phases. The acute infection is presented with blood parasitemia and is often mildly symptomatic. Infected individuals then evolve into chronic phase. While many remain in an indeterminate phase without any clinical symptoms, ~30% progress to develop clinically relevant Chagas disease. Chronic Chagasic Cardiomyopathy is a complex disease that includes a wide-spectrum of manifestations, ranging from minor myocardium involvement to left ventricular (LV) systolic dysfunction, dilated cardiomyopathy, arrhythmias, thromboembolic events, and terminal cardiac failure [1]. Gastrointestinal (GI) manifestations, such as mega-syndromes involving tubular structures of the GI tract, though not commonly recorded, are frequent in certain geographic areas [2].

The vectorial transmission of the parasite by hematophagous triatomines (kissing bugs) is the most commonly recognized route of infection in endemic areas [3], though other routes of infection including transfusion of contaminated blood [4] and transplantation of infected organs [5] have also been noted. Congenital transmission (CT) of *T*. *cruzi* is also an important public health problem [6]. Though underestimated and underreported, recent estimates indicate that congenital transmission of *T*. *cruzi* occurs at a rate of 1–12% in endemic Latin American countries [7–9]. If left untreated, neonates may develop cardiac or enteric disease at delivery or weeks’ later [10]. Severity of the disease in infected children varies greatly from mild symptomatic cases to fatal cases [11,12]. Several factors can be involved in CT of *T*. *cruzi* and disease development; among them the genetic variability of the parasite may have considerable effect [13,14].

The CT of *T*. *cruzi* can occur at any time during gestation [15], though some investigators have suggested that maximal likelihood of congenital infection occurs during the third trimester of gestation [10] when the placenta undergoes a series of physiological and metabolic transformations that favor the invasion of infectious agents [16,17]. Trophoblastic detachment [18] and apoptosis [19] with disorganization of basal lamina and collagen destruction [20,21] in placenta of woman with chronic Chagas disease is documented. Studies of CT of *T*. *cruzi* in rats and mice demonstrated the presence of parasite in the gravid uterus and amniotic fluid [10].

Guinea pigs (*Cavia porcellus*) are an important natural reservoir host for *T*. *cruzi*, and mimic reproducible and comparable human phases of acute infection and chronic disease [22,23]. Importantly, guinea pigs share several anatomical aspects with the placenta of humans since there is an extensive invasion of the trophoblast (hemo-monocortical placentation) [24,25]. Because of their longer time of gestation compared with murine models and a potentially higher surface of contact of the pathogen with the placenta, guinea pigs offer an excellent model for the study of congenital transmission of *T*. *cruzi* infection.

In this study, our main goal was to establish the experimental guinea pig model of congenital transmission of *T*. *cruzi*. We also measured the effects of *T*. *cruzi* exposure at various stages of gestation on fertility, pregnancy outcome, and heart and colon pathology in mother and pups. We discuss the potential utility of guinea pig model in screening new drugs and vaccines against CT of *T*. *cruzi*.

## Materials and methods

### Ethics statement

All animal experiments were performed according to the National Institutes of Health Guide for Care and use of Experimental Animals, and approved by the bioethics committee at the Campus of Biological and Agricultural Sciences, Autonomous University of Yucatan (No. CB-CCBA-M-2016-001). The maintenance of the animals was performed under the current official Mexican standards (NOM-062-ZOO-1999), “Technical specifications for the production, care and use of laboratory animals.”

### Animals, parasites, and infection

Female SPF guinea pigs (*Cavia porcellus*) weighing 650 ± 50 g (4–5 months old) were purchased from The Mexican Center for Research and Advanced Studies of the National Polytechnic Institute (CINVESTAV), and confirmed seronegative for *T*. *cruzi* by two tests before use in this study. Animals were kept in individual cages under controlled conditions of temperature, humidity and light cycles according to the requirements of the species, with food and water *ad libitum*. Pregnancy was diagnosed by day 25 post-mating using an ultrasound with a micro-convex 7.5 MHz transducer.

**Fig 1:**
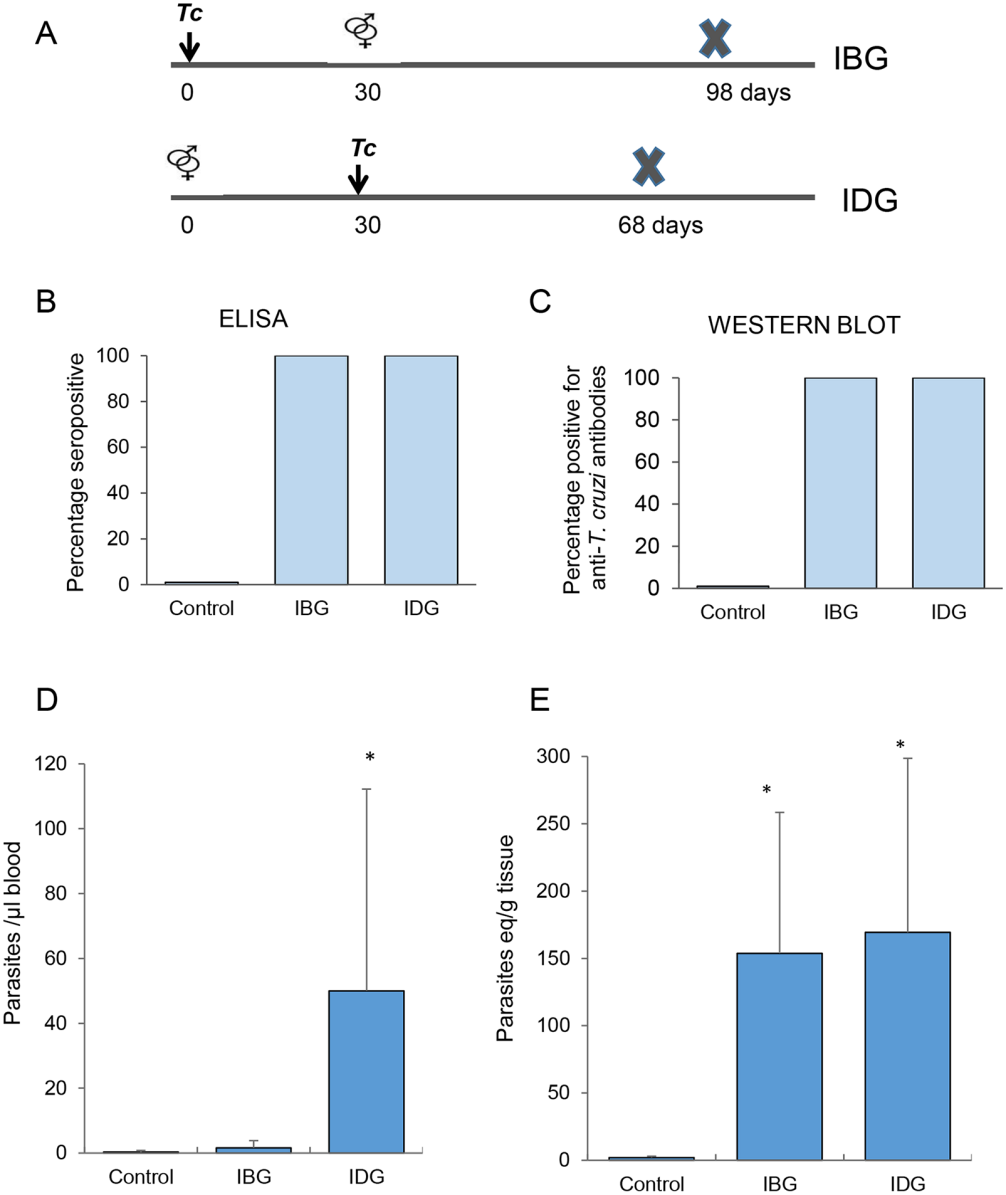
Serological and parasitological analysis of guinea pig dams infected with *T*. *cruzi*. **(A)** Chronogram of female guinea pigs infected before gestation (IBG) and infected during gestation (IDG). The time-points (days) of breeding (⚤), *T*. *cruzi* (*Tc*) inoculation (black arrow), and euthanasia (marked by X) are shown. Females that were mated but not infected were used as controls. **(B&C)** An ELISA **(B)** and Western blotting **(C)** were performed to monitor the anti-T. *cruzi* antibodies in sera samples of control, IBG and IDG dams at parturition. Bar graphs show the percentage of dams that were positive for anti-*T*. *cruzi* antibodies. **(D&E)** Quantitative PCR evaluation of blood **(D)** and heart tissue **(E)** parasite load in control, IBG and IDG dams is shown. * P<0.05.

*T*. *cruzi* H4 strain of TcI lineage [26] was propagated by *in vitro* passage in C2C12 cells. Three study groups were established with five female guinea pigs per group. Animals were inoculated with 100 trypomastigotes of H4 strain via intraperitoneal (IP) route. Female guinea pigs in Group A were infected, and 30 days later mated with males. Infection before gestation (IBG) was performed to study CT in indeterminate phase of infection. Animals in Group B were mated, and at mid-term of pregnancy (i.e., 30 ± 3 days of gestation), infected with *T*. *cruzi*. Infection during gestation (IDG) was performed to study the effect of acute infection during pregnancy on CT. Animals in Group C were mated but not infected, and used as a control group. Line diagram of infection and mating schedule is presented in Fig 1A.

### Sampling

Animals from all groups were euthanized at the end of the pregnancy according to the official Mexican standards (NOM-033-SAG/ZOO-2014), “Methods for killing domestic and wild animals.” The prolificacy (total number of fetuses obtained by each female per gestation) and the size and weight of fetuses were recorded. Likewise, macroscopic alterations in fetuses indicating the location and severity of damage were recorded. Fetal tissue sections (heart, liver, skeletal muscle, and spleen) were fixed in 10% buffered formalin for histology and stored at -20°C for DNA purification. Blood samples were taken from the dams and fetuses for isolating DNA or for collecting the serum, and all samples were stored at -20°C until analysis.

### Serology

Sera samples were monitored for *T*. *cruzi*-specific IgG antibody response by an enzyme-linked immunosorbent assay (ELISA) by using a Wiener Chagatest-ELISA recombinant v.4.0 kit. The kit detects antibody response to six recombinant proteins that are expressed in *T*. *cruzi*. The assay was carried out following the manufacturer's recommendations, except that 2^nd^ antibody was replaced with goat anti-guinea pig IgG conjugated with HRP (sc2903, Santa Cruz Biotechnology, Dallas TX). Briefly, 96-well plates were coated with recombinant proteins, and then sequentially incubated with 20-μl sera samples (1:100 dilution) and HRP-conjugated guinea pig anti-IgG (1: 5000 dilution) diluted in phosphate buffer (137 mM NaCl, 2.7 mM KCl, 4.3 mM Na_2_HPO_4_, and 1.4 mM KH_2_PO_4_, pH 7.4). The color was developed with tetramethylbenzidine and hydrogen peroxide substrates, and reaction was stopped by acidification of the reaction medium. The optical density was recorded at 450 nm by using an xMark microplate absorbance spectrophotometer (Bio-rad, Hercules, CA). The cut-off was determined from the mean value of the negative control sera samples ± 3 standard deviations. The sensitivity and specificity of the test was recorded at 99% and 98.3%, respectively.

### Western blotting (WB) and Indirect Immunofluorescence assay (IFA)

Western blotting (WB) and Indirect Immunofluorescence assay (IFA) have been previously described by us [27]. Briefly, epimastigotes of H4 strain parasites were washed in PBS, lysed with Laemmli sample buffer containing protease inhibitor cocktail (Sigma, St. Louis, MO), and protein samples (20 μg) were resolved on 10% polyacrylamide gels. Proteins were wet-transferred to nitrocellulose membranes, and membranes were blocked for 30 min with 1% non-fat milk in Tris-Buffered Saline—Tween 20 (TBST; 10 mM Tris HCl, 150 mM NaCl, 5 mM Tween 20, pH 8.0), and incubated overnight at 4°C with polyclonal serum from infected and non-infected animals (1:100 dilution in TBST-1% non-fat milk). Membranes were then incubated for 1 h with alkaline phosphatase—conjugated secondary antibody and color was developed with nitro-blue tetrazolium chloride and 5-bromo-4-chloro-3’-indolyl phosphate p-toluidine salt (NBT and BCIP, respectively). All sera samples were analyzed in triplicate, and a serum sample was considered positive when it recognized at least five antigenic bands; the results were considered indeterminate when the sample recognized one to four antigenic bands, and negative when the serum sample showed no reactivity.

For IFA, H4 epimastigotes were suspended in PBS (5x10^6^ parasites/mL), and added to 8-well glass slides (10 μl/per well). Slides were air-dried for 2 h, blocked for 30 minutes with PBS-5% horse serum (PBS-HS), and incubated for 45 min sera samples from guinea pigs (1:32 to 1:2048 in PBS-HS). Slides were then incubated for 45 min with FITC-conjugated secondary antibody (1:80 dilution in PBS-HS, from Sigma) and 0.01% Evan’s blue. Finally, slides were covered with Vectashield (Vector Laboratories, Burlingame, CA) mounting medium and microscopically examined using a Nikon TE300 (Nikon, Tokyo, Japan) microscope. Sera samples exhibiting high anti-IgG antibodies titer by IFA at 1: 32 dilution (or more) were considered positive.

### DNA extraction

The DNeasy Blood and Tissue Kit (69504, Qiagen, Germantown MD) was used to isolate the genomic DNA from the blood and tissue samples by following the manufacturers’ instructions. Total DNA was examined for quality (OD_260_/OD_280_ ratio of 1.7–2.0) and quantity ([OD_260_ –OD_320_] x 50-μg/ml) by using a DU 800 UV/visible spectrophotometer. All samples were evaluated by quantitative PCR (qPCR) for glyceraldehyde-3-phosphate dehydrogenase gene (GAPDH) to document the absence of any kind of inhibitors in the reaction mix and to establish equal amounts of DNA samples are used for all reactions as described.

### Quantitation of parasite burden

The parasite load in all samples was measured by using a SYBR Green-based quantitative PCR as described [28]. Briefly, total DNA (25 ng) was used as template with 0.5 μM of *T*. *cruzi* nuclear satellite DNA-specific oligonucleotides (TCZ-F 5'-GATCTTGCCCACAMGGGTGC-3' and TCZ-R 5'-CAAAGCAGCGGATAGTTCAGG-3') and SsoAdvanced Universal SYBR Green Supermix (172–5271, Bio-Rad) in a final volume of 20 μl. The cycling parameters were one step of 15 min of denaturation at 95°C; 50 cycles of PCR amplification (95°C for 10 s, 55°C for 15 s and 72°C for 10 s). Fluorescence data collection was performed at 72°C at the end of each cycle. After quantification, a melt curve was made with 74–85°C rising by 0.5°C each step and waiting for 4 seconds afterwards acquiring data on Green channel. Melting temperature (Tm) of the amplicon was 81°C. Finally, data were analyzed with CFX Manager Software V 2.1.

Standard curve was prepared by using epimastigotes of *T*. *cruzi* DTU VI (CL Brener), as described [29]. Briefly, 200 μl of blood from a seronegative *C*. *porcellus* was spiked with 10^7^ parasites/ml, and total DNA was extracted. Ten-fold dilutions of the extracted DNA, corresponding to 10^6^–0.1 parasite/ml, were used in qPCR, as above. Likewise, different standard curves were prepared for evaluation of parasites in placental, heart, skeletal muscle, and spleen tissues. Total DNA extracted from blood and tissues of uninfected guinea pigs were used as negative controls. All samples were tested in triplicate, and parasite standard curve, negative controls, and no-template DNA control were included in all qPCR experiments. The data are presented as parasite equivalent/μL blood or parasite equivalent/g tissue, respectively.

### Histology

Heart tissue sections of female guinea pigs and fetal tissue samples (utero-placental unit, heart and skeletal muscle) were fixed in 10% buffered formalin for 24 hours, dehydrated in absolute ethanol, cleared in xylene, and embedded in paraffin. Five-micron tissue sections were mounted on glass slides, deparaffinized, and subjected to Hematoxylin & Eosin staining. In general, we analyzed each tissue-section for randomly selected, 10-microscopic fields (100X magnification), and examined three different tissue sections/organ/animal. The tissue sections were evaluated by two individuals in a blinded manner. The presence of inflammatory cells in H&E stained sections was scored as 0 (absent), 1 (focal or mild, 0–1 foci), 2 (moderate, ≥2 foci), 3 (extensive inflammatory foci, minimal necrosis, and retention of tissue integrity), and 4 (diffused inflammation with severe tissue necrosis, interstitial edema, and loss of integrity) [30]. Inflammatory infiltrates were characterized as diffused or focal depending upon how closely the inflammatory cells were associated. Tissue (muscle) degeneration was qualified based on the presence of swelling, fragmentation and rupture of fibers, loss of striation, and/or vacuolation.

### Statistical analysis

All experiments were conducted at least twice (n = 5 guinea pigs/group/experiment), and all samples were analyzed in triplicate. Data are expressed as mean ± standard deviation (SD). Comparisons of means between and within groups were performed using the Student's t test and ANOVA, considering a significance of 95% (i.e., p <0.05). The CT was considered when amastigote nests or *T*. *cruzi* DNA were detected in any of the fetal tissues and histological lesions were observed.

## Results

**Fig 2:**
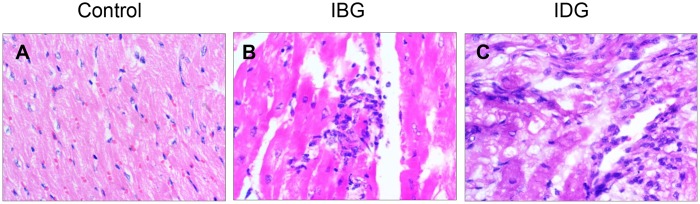
Histological evaluation of the heart of dams. Guinea pigs were infected with *T*. *cruzi* before (IBG) or during (IDG) gestation, and harvested close to parturition, as described in Materials and Methods and Fig 1A. Shown are the representative images of the H&E staining of the heart tissue sections from **(A)** Control, **(B)** IBG, and **(C)** IDG dams. Semi-quantitative score of inflammatory infiltrate is presented in Table 1.

All dams in IBG and IDG groups were seropositive for *T*. *cruzi*-specific antibodies at the end of the study. The sera levels of IgG antibodies, monitored by an ELISA (Fig 1B) and Western blotting (Fig 1C), were similar in dams of both infected groups. Infection by *T*. *cruzi* was also confirmed by qPCR detection of parasites in blood and heart tissue of the dams. Dams in IBG group exhibited low but detectable blood parasites, and a high variation in the blood parasitic load was observed in dams of the IDG group (Fig 1D). Yet, dams of IDG group exhibited >40-fold higher level of blood parasitemia that was statistically significant (p<0.05) when compared to that detected in IBG dams (Fig 1D). Heart tissue parasites were detected at similar level in all infected dams (Fig 1E). Histological evaluation of the heart tissue of dams from the IBG and IDG groups showed inflammatory infiltrate with necrosis of the cardiac myocytes (Fig 2B & 2C). The extent of tissue necrosis was slightly more severe in the IDG group (Table 1) where cytoplasmic vacuoles, indicative of hypoxic tissue degeneration, were also noticed. Fiber degeneration evidenced by disorganized/fragmented striations was also seen in heart tissue of infected dams in the IBG and IDG groups (Table 1). No inflammatory infiltrate, necrosis or muscle degeneration was noted in the heart tissue of control dams (Fig 2A). Together the results presented in Figs 1 and 2 suggest that a) *T*. *cruzi* H4 isolate is virulent, and it replicates and disseminates to tissues in guinea pigs, b) acute parasitemia is presented by IDG dams, and c) indeterminate phase of infection is presented in IBG guinea pigs. Further, tissue parasite burden resulted in infiltration of inflammatory infiltrate and heart tissue necrosis in all infected dams.

**Table 1:** Frequency and severity of histological lesions in guinea pig dams and their fetuses in response to infection by *Trypanosoma cruzi*.

Dams tissue	Group	Inflammatory infiltrate		Cellular necrosis	Fiber degeneration	Amastigotes nest
		# of dams affected (%)	Score	# of dams affected (%)		
Heart	CG	0/5 (0%)	0/5 (0%)	0/5 (0%)	0/5 (0%)	0/5 (0%)
	IBG	5/5 (100%)	1.2±0.4	7/12 (58.3%)	5/12 (41.6%)	0/5 (0%)
	IDG	5/5 (100%)	1.6±0.5	6/12 (50%)	8/12 (66.6%)	0/5 (0%)
Fetal tissue	Group	Inflammatory infiltrate		Cellular necrosis	Fiber degeneration	Amastigotes nest
		# of fetuses affected (%)	Score	# of fetuses affected (%)		
Heart	CG	0/18 (0%)	0	0/18 (0%)	0/18 (0%)	0/12 (0%)
	IBG	3/12 (25%)	1.0±0	7/12 (58.3%)	5/12 (41.6%)	0/12 (0%)
	IDG	2/12 (16.6%)	1.5±0.7	6/12 (50%)	8/12 (66.6%)	0/12 (0%)
Skeletal muscle	CG	0/18 (0%)	0	0/10 (0%)	0/10 (0%)	0/10 (0%)
	IBG	2/9 (22.2%)	1.0±0	3/9 (33.3%)	4/9 (44.4%)	0/9 (0%)
	IDG	1/11 (9%)	1.0±0	7/11 (63.6%)	6/11 (54.5%)	0/9 (0%)
Placenta	Group	Inflammatory infiltrate		Dystrophic calcification	Not applicable	Amastigotes nest
		# of fetuses affected (%)	Score	# of fetuses affected (%)		# of fetuses affected (%)
	CG	0/12 (0%)	0	12/12 (100%)		0/12 (0%)
	IBG	0/12 (0%)	0	12/12 (100%)		0/12 (0%)
	IDG	0/12 (0%)	0	12/12 (100%)		0/12 (0%)

Female guinea pigs were infected before gestation (IBG) or during gestation (IDG) as described in Fig 1A. Control group (CG) included females that were mated but not infected. Females were euthanized towards the end of pregnancy. Dams and fetal tissue sections from were stained with H&E (two sections per tissue per animal), and analyzed by light microscopy (>10 microscopic fields per tissue section). Semi-quantitative score for inflammatory infiltrate was calculated as: (0) Absent, (1) Focal or mild, 0–1 foci, (2) Moderate ≥2 foci, (3) extensive inflammation foci, minimal necrosis, and retention tissue integrity, (4) Diffuse inflammation with severe tissue necrosis, interstitial edema and loss integrity.

**Fig 3:**
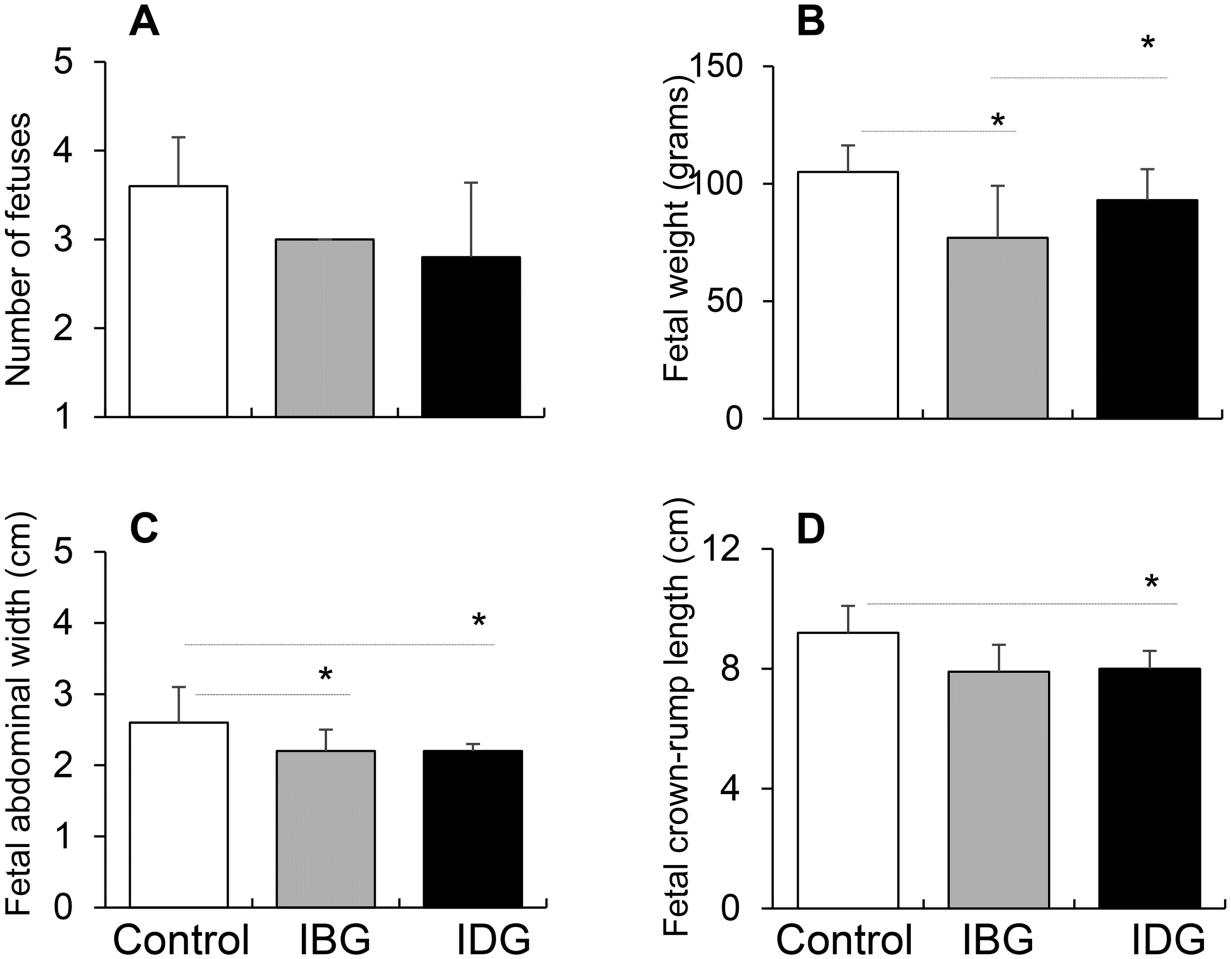
Effect of T. *cruzi* infection on fetal growth. Female guinea pigs were infected before gestation (IBG) or during gestation (IDG) as shown in Fig 1A. Guinea pigs that were mated but not infected were used as controls. Fetal characteristics, including number of fetuses carried per guinea pig **(A)**, fetal weight **(B)**, fetal abdominal width **(C)** and fetal crown-rump length **(D)** were monitored close to parturition. Data are presented as mean value ± SD, and significance (* P < 0.05, control vs. infected) is plotted.

Several mating attempts were needed to get the dams in infected groups (IBG and IDG) pregnant, while all control females became pregnant in first attempt. Once pregnant, no statistically significant differences were observed in the number of fetuses carried by females in different groups. The average number of fetuses carried by IBG and IDG females were 2.8 ± 0.8 and 3.0 ± 0.0 respectively, and comparable to that noted in control group (Fig 3A). However, significant differences were observed in the overall growth of the fetuses of the IBG and IDG females when compared to the control group. Fetuses carried by IBG (vs. control) females were 26%, 14%, and 15% smaller in weight, length, and width, respectively (all, p<0.05), while fetuses carried by IDG (vs. control) females were 11% and 15% lesser in weight and width (p<0.05), and 13% lower in length (p>0.05) (Fig 3B–3D). These results suggest that *T*. *cruzi* infection of dams before or during gestation results in decreased frequency of pregnancy and decreased growth of the fetuses.

**Fig 4:**
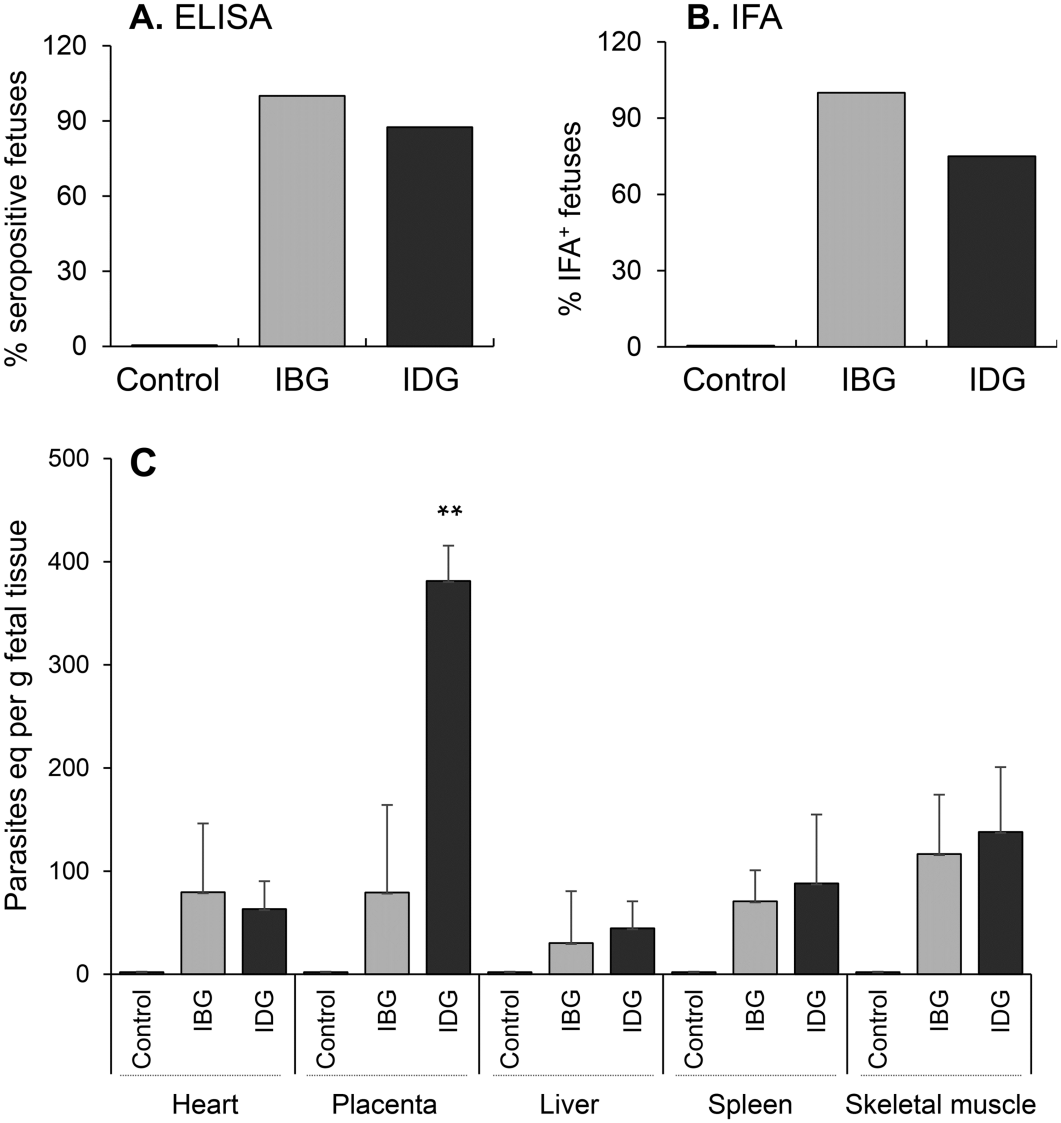
Serological and parasitological analysis of fetuses of guinea pigs infected with *T*. *cruzi*. Female guinea pigs were infected before gestation (IBG) or during gestation (IDG). **(A&B)** Sera samples of fetuses were obtained close to parturition, and analyzed by an ELISA ***(A)*** and indirect immunofluorescence assay **(B)**. Bar graphs show the percentage of fetuses in each group that were positive for anti-*T*. *cruzi* antibodies. **(C)** Fetal tissue parasite load was monitored by quantitative PCR as described in Materials and Methods. Control (n = 9); IBG (n = 9); IDG (n = 5) samples were analyzed in triplicate. Data in panel A & B are presented as absolute values, and data in panel C are presented as mean value ± SD. (**P <0.01, IDG vs. IBG).

Next, we determined if differences in fetal size correlates with congenital transmission of *T*. *cruzi*. All sampled fetuses were seropositive for *T*. *cruzi*-specific IgG antibodies by an ELISA (Fig 4A) and IFA (Fig 4B). A quantitative measure of tissue parasite load in fetuses was obtained by qPCR (Fig 4C). The qPCR data showed that all fetuses carried by dams in IBG and IDG groups were infected. Tissue parasite load in fetal tissues of IBG and IDG groups was observed in the order of skeletal muscle > heart > spleen > liver. Parasite load in fetal heart, spleen and skeletal tissue did not show statistical differences between IBG or IDG groups. However, placental parasite burden in fetuses of IDG dams was 4-fold higher than that observed in placental tissue of IBG dams (Fig 4C). Together, these results suggest that maternal-fetal transmission of *T*. *cruzi* occurred at high rate in guinea pigs exposed to the parasite before or during gestation. Exposure during gestation poses higher risk of congenital transmission because of high parasitemia in placental tissue.

**Fig 5:**
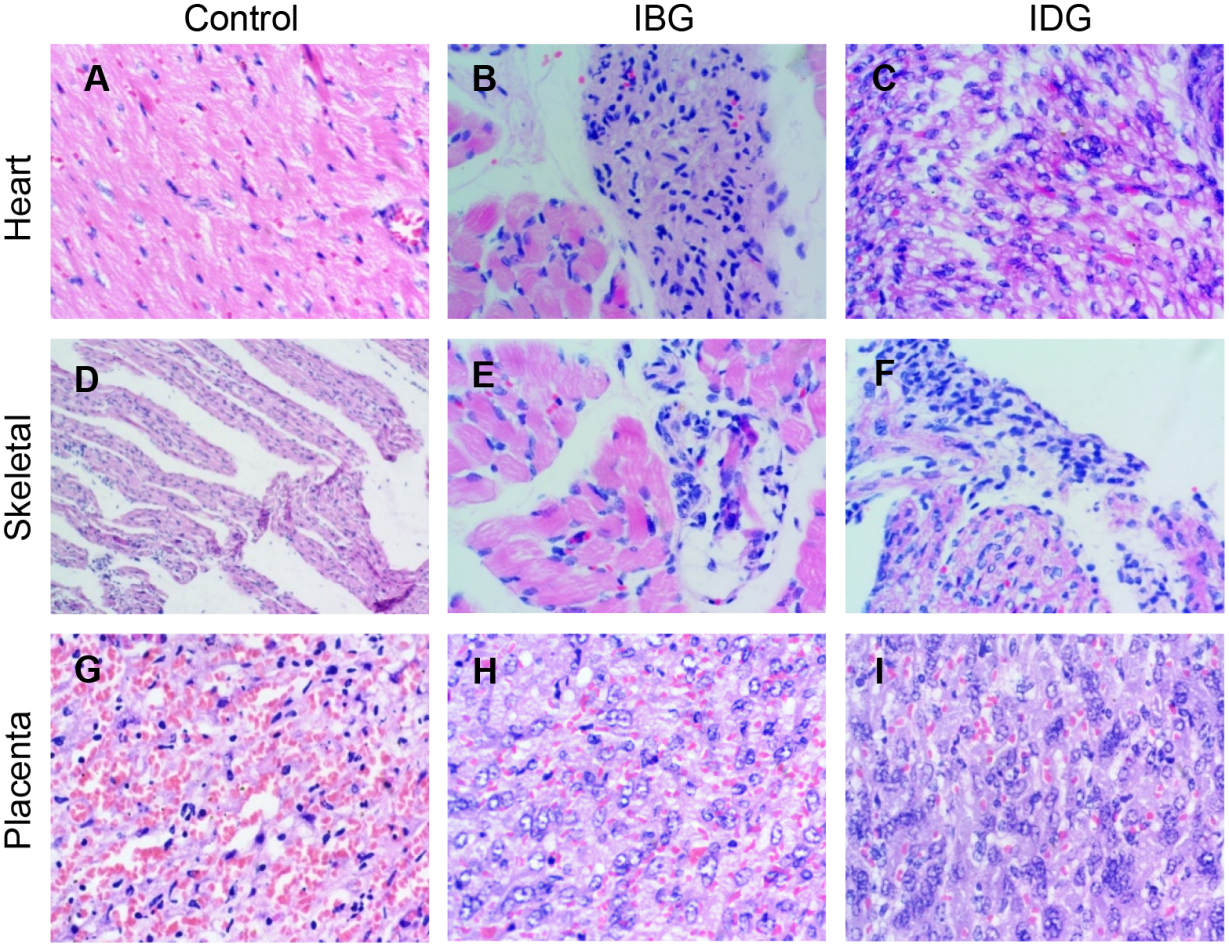
Histological evaluation of the fetal tissues. Guinea pigs were infected with *T*. *cruzi* before (IBG) or during (IDG) gestation, and harvested close to parturition, as described in Materials and Methods and Fig 1A. Guinea pigs that were mated but not infected were used as controls. Shown are the representative images of the H&E staining of the fetal heart **(A-C)**, fetal skeletal muscle **(D-F)**, and fetal placenta **(G-I)** carried by control **(A, D, G)**, IBG **(B, E, H)**, and IDG **(C, F, I)** dams. Semi-quantitative score of inflammatory infiltrate observed in fetuses is presented in Table 1.

Finally, fetal tissues were evaluated by histology for *T*. *cruzi* induced inflammation and tissue damage. Representative images of H&E stained fetal tissue sections (heart, skeletal muscle and placenta) are presented in Fig 5. Frequency and severity of lesions in different tissues of fetuses from both infected groups compared with the control group is presented in Table 1. No amastigote nests were detectable by histological analysis of tissue sections (heart, placenta and skeletal muscle) of fetuses carried by dams in IBG and IDG groups. The heart of fetuses from the IBG group showed moderate inflammatory infiltrate with cellular necrosis in 25–58% of infected fetuses, whilst in the IDG group more severe lesions were detected in 16.6 to 50% of infected fetuses (score 1.5±0.7), and characterized by multifocal necrosis of myocytes and degeneration seen as multiple intra-cytoplasmic vacuoles (Fig 5B & 5C, Table 1). The skeletal muscle of fetuses from both IBG and IDG groups showed infrequent interstitial edema with lymphocyte inflammatory infiltrate scored as 1 (Fig 5E & 5F, Table 1). No pathological changes were noted in the placenta of the dams in control as well as infected groups (Fig 5G–5I). Only structural changes of the placentas, such as dystrophic calcification, that are commonly observed in normal advanced gestations, were seen in all groups. These results suggest that all fetuses carried by infected dams are at risk of *T*. *cruzi* induced tissue damage. The extent of fetal tissue damage is more pronounced when the parasite exposure occurs during pregnancy and results in increased parasitemic status. The placental tissues, despite the presence of high degree of parasite burden, are protected from infection induced cellular damage.

## Discussion

Results of the present study demonstrate the high capacity of congenital transmission of *T*. *cruzi* strain H4 (DTU I) in a guinea pig model even when using a low dose of inoculum. Guinea pigs, like humans, have a deep invasion of the trophoblast into the maternal decidua, which is limited in most other rodent models [31], and guinea pigs mimic the human pregnancy by the progesterone profile; progesterone is produced in the placenta by the end of gestation that does not decline at term [32] contrary to what happens in most rodents and other mammals. Further, the long gestation period of 72 days provides an opportunity to study the effect of parasite replication in dams during gestation on fetal development. Thus, guinea pigs offer the best available model for studying the human congenital infection.

Cencig et al, [33] observed a very low rate of congenital infection, occurring in approximately 4% of living pups born to acutely infected mice. In another study, Wistar rats acutely infected with *T*. *cruzi* (DTU II) also exhibited a very low rate of parasite congenital transmission though a substantial decline in weight and an increase in acute myocarditis was noted in the pups that were congenitally infected [34]. Mjihdi et al, [35] observed infertility and fetal death in mice infected with a *T*. *cruzi* DTU I strain. The authors noted a massive invasion of the decidua, ischemic necrosis of placental tissue, poor fetal growth, and significant fetal losses in mice; however, no evidence of congenital transmission in fetuses was noted. Likewise, Badra [36], Sala [37] and coworkers, evaluating the effect of *T*. *cruzi* strains in pregnant mice, observed a decline in fetal weight and length, placental weight and umbilical cord length, but no or low frequency of infection of the fetuses. Altogether, these studies indicate that fetal loss and a decline in fetal growth in mice occurs due to placental infection and infection induced pathologies in the placental tissue. Yet, mice did not provide a true indication of congenital infection of pups. Our guinea pig model captured all the benefits that can be useful in studying the efficacy of therapies against congenital transmission. One, all guinea pig dams, irrespective of their exposure to infection before or during gestation, carried fetuses that were congenitally infected by *T*. *cruzi*, and these fetuses also exhibited growth retardation and histological lesions in placental, cardiac and skeletal tissues. Two, with inoculation of low dose of parasite, all dams still became infected, and a decline in number of fetuses carried by infected (vs. normal) dams, still-birth, or fetal reabsorption was not observed at a significant level. Thus, it was feasible to obtain power for analyzing the effect of congenital infection in small number of guinea pig dams. Three, all fetuses of infected dams were serologically positive indicating the trans-placental transfer of maternal antibodies and functionality and transfer capacity of the placenta in guinea pigs, as is reported in human newborns of infected mothers [38]. Thus, we surmise that guinea pigs provide a true model of maternal-fetal transmission of *T*. *cruzi* and Chagas disease, and also offer opportunity for efficacy testing of therapies to prevent fetal infection.

There is a high correlation between fetal heart rate in early pregnancy and the crown-rump length of the human neonates [39]. When first trimester embryos develop a low heart rate, there is a dismal prognosis of pregnancy resulting from embryo underdevelopment [40]. Alterations in the blood flow due to reduced heart rate and cardiac output is shown to affect the growth and development of chicken embryos [41]. *Trypanosoma cruzi* (DTU TcI) is able to produce high parasitemia rates and because of their high tropism to cardiac tissue during the acute and chronic phases of the disease can lead to myocarditis, lymphocyte and monocytes infiltrations and production of amastigote nests [42]. In the present study a high tropism towards cardiac tissue of the dams and damage of the cardiac tissue of fetuses was seen where the parasites probably replicated leading to impaired function and affecting the embryo viability and growth.

Placenta is the key fetal organ responsible for protecting the fetus from any infectious pathogen [15]. Congenital transmission of *T*. *cruzi* is suggested to occur when the phagocytic capacity of the placenta is compromised [43] and a significant amount of blood parasites are present in the infected women [44]. Others have suggested that the maternal-fetal transmission of *T*. *cruzi* occurs when parasites cross the chorionic plate at the marginal sinus where the trophoblastic covering is incomplete. This is considered as a “sensible zone” where infected fibroblasts and macrophages can potentially transport trypomastigotes to a fetus. Reported lesions in the placenta induced by *T*. *cruzi* include inflammatory processes of the chorion with infiltration of polymorphonuclear immune cells and umbilical edema. However, lesions in the placenta are discrete, not very specific and with a low number of parasites so the placental examination is not a good diagnostic tool for the maternal-fetal transmission of Chagas disease [45]. In our model, we did not detect evidence of placental damage in the infected dams, and we also did not see a strong correlation between the qPCR detection of parasites in blood or placental tissue to transmission of parasite to fetuses. Thus, the route of transmission to the embryos is not clearly established. We suspect that due to the low dose of the parasite used, the placental response and rearrangement was not produced, and hematogenous trans-placental transmission occurred silently. Another probable route of fetal infection is through the invasion of marginal zones of the placenta, which is devoid of trophoblasts, and thus allow the passage of parasites especially during a moderate parasitemia. A trans-uterine route of transmission has also been suggested [15], but it is less probable and difficult to probe.

Parasite is primarily detected in the heart during the chronic phase of the disease. However, tropism of *T*. *cruzi* is not exclusive to cardiac tissue [46]. Other organs such as kidney, lung, pancreas, gastrointestinal tissue, skeletal muscle, spleen, and liver etc. are normally infected during experimental acute infection in mouse models [47]. Our finding of *T*. *cruzi* in multiple fetal organs suggests that pups develop an acute-like infection after exposure to the parasite from mother. It is likely that parasite replication occurs in the placenta, and when infected placental cells release the trypomastigotes, the parasite reaches fetus through the trophoblast or aspiration of amniotic fluid by the fetuses, and then spreads by active penetration through skin, mucosal membranes or organ-to-organ dispersion [48,49]. The degree of invasion to fetal tissues and disease severity may also depend on the virulence and dose of the strain of *T*. *cruzi* involved [50,51]. In the present study, a very low dose (100 parasites) of a high virulent strain of *T*. *cruzi* was able to invade diverse tissues of the fetuses during the congenital transmission.

In summary, we have demonstrated a guinea pig model of *T*. *cruzi* infection and maternal-fetal transmission. Even very low dose of *T*. *cruzi* DTU I was sufficient to establish high rate of congenital transmission to fetuses. The frequency of transmission to fetuses was high irrespective of whether the mother was exposed to infection before or during gestation, though the extent of fetal tissue damage tended to be higher when dams were exposed to parasite during gestation. Guinea pigs share several anatomical aspects with the human placenta and have a longer gestation than other rodent models, and thus offer an excellent model for the study of congenital transmission of the diverse linages of *T*. *cruzi*. Our findings also call for *T*. *cruzi* screening in pregnant women and adequate follow up of the newborns in endemic areas.
